# Modification of Daly’s Do-it-yourself, Ultrasound-guided Pericardiocentesis Model for Added External Realism

**DOI:** 10.5811/westjem.2018.2.37736

**Published:** 2018-03-13

**Authors:** Timothy P. Young, Heather M. Kuntz

**Affiliations:** Loma Linda University Medical Center and Children’s Hospital, Department of Emergency Medicine, Loma Linda, California

Comment on:

Daly R, Planas JH, Edens MA. Adapting gel wax into an ultrasound-guided pericardiocentesis model at low cost. *West J Emerg Med*. 2017;18(1):114–6.

We were happy to discover the article by Daly and colleagues describing a low-cost, pericardiocentesis training model (Figure 1).^1^ We have struggled to find a cost-effective means of demonstrating and practicing ultrasound-guided pericardiocentesis with our emergency medicine residents. We greatly appreciated their ingenuity in building upon and improving previous do-it-yourself models.^2^,^3^ We were especially impressed with their addition of a plastic Halloween skeleton thorax and 250 mL normal saline bag to act as the pericardial sac. In that same spirit, we have devised a modification of their model that offers the benefit of more-realistic external landmarks.

We followed their instructions with several exceptions. Instead of the square-shaped plastic container that they used for a chest wall mold, we used a plastic, manikin dress-form torso (Amazon, $25; https://tinyurl.com/amazondressform). We cut the back of the dress form out with tin snips and turned it upside down to act as a mold. We also used ballistics gel (Amazon, $60; https://tinyurl.com/amazonballisticsgel) instead of gel wax. Ballistics gel does not require the addition of a substance such as flour to simulate the echogenicity of human tissue. Ballistics gel is clear, so we added an optional flesh-colored dye (Humimic, $30; https://humimic.com/product-category/dye/). Like wax gel, ballistics gel can be removed and re-melted to create a new model after repeated needle aspirations. We melted the ballistics gel in a Hamilton-Beach 7-quart cooker (Amazon, $30; https://tinyurl.com/amazonHBcooker) for several hours on the “high” setting, then stirred in the dye and poured the ballistics gel into the turned-over torso (Figure 2). Unlike the Daly model, we did not need to add flour or strain off foam. We placed ice underneath the torso to prevent melting or deforming of the hard plastic torso shell, but this may have been unnecessary.

Like the Daly model, we were able to build our model for less than $200. The additional benefit we found was that our model created a realistic chest surface and external landmarks. Our residents were able to practice ultrasound-guided sub-xiphoid and parasternal approaches as well as the blind, landmark-based pericardiocentesis technique. Training programs planning to utilize Daly’s brilliant innovation may wish to follow our lead to further increase realism.

## Figures and Tables

**Figure 1:**
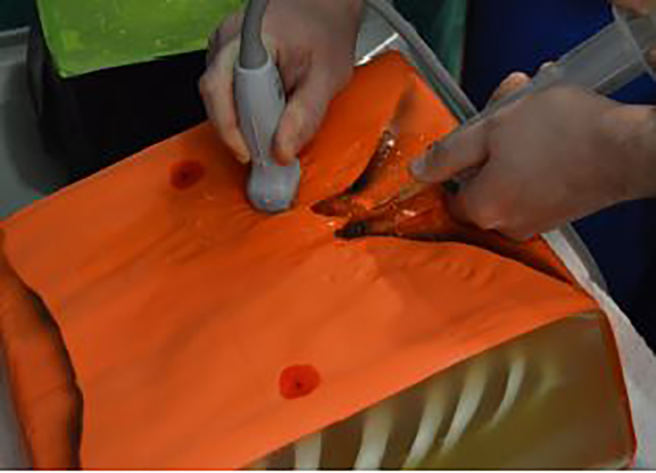
Original ultrasound-guided pericardiocentesis model from Daly R. et al. *West J Emerg Med.* 2017;18(1):114–6.

**Figure 2:**
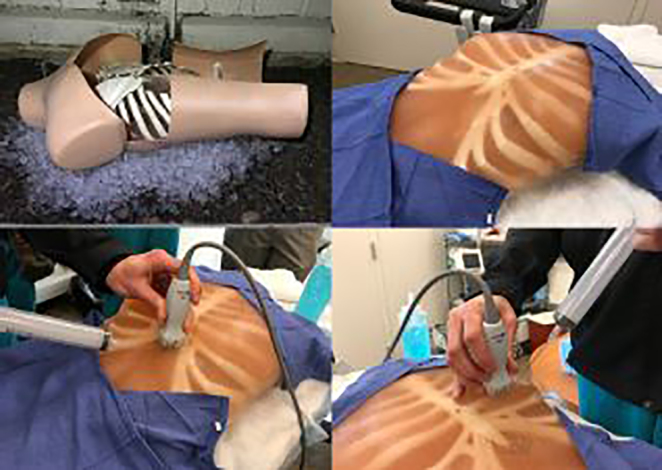
Modified ultrasound-guided pericardiocentesis trainer using a plastic dress form torso turned upside down to act as a mold.
